# Dimerization of ABCG2 Analysed by Bimolecular Fluorescence Complementation

**DOI:** 10.1371/journal.pone.0025818

**Published:** 2011-10-03

**Authors:** Ameena J. Haider, Deborah Briggs, Tim J. Self, Hannah L. Chilvers, Nicholas D. Holliday, Ian D. Kerr

**Affiliations:** School of Biomedical Sciences, University of Nottingham, Nottingham, United Kingdom; University of Cambridge, United Kingdom

## Abstract

ABCG2 is one of three human ATP binding cassette transporters that are functionally capable of exporting a diverse range of substrates from cells. The physiological consequence of ABCG2 multidrug transport activity in leukaemia, and some solid tumours is the acquisition of cancer multidrug resistance. ABCG2 has a primary structure that infers that a minimal functional transporting unit would be a homodimer. Here we investigated the ability of a bimolecular fluorescence complementation approach to examine ABCG2 dimers, and to probe the role of individual amino acid substitutions in dimer formation. ABCG2 was tagged with fragments of venus fluorescent protein (vYFP), and this tagging did not perturb trafficking or function. Co-expression of two proteins bearing N-terminal and C-terminal fragments of YFP resulted in their association and detection of dimerization by fluorescence microscopy and flow cytometry. Point mutations in ABCG2 which may affect dimer formation were examined for alterations in the magnitude of fluorescence complementation signal. Bimolecular fluorescence complementation (BiFC) demonstrated specific ABCG2 dimer formation, but no changes in dimer formation, resulting from single amino acid substitutions, were detected by BiFC analysis.

## Introduction

Resistance of cancers to a chemically broad spectrum of drugs is referred to as multidrug resistance (MDR). Among the many factors influencing MDR in humans are three members of the ATP binding cassette (ABC) transporter family [1]. The ABCB1 (P-glycoprotein) and ABCC1 (multidrug resistance protein-1) are both likely to act as monomeric proteins since they contain the four domains expected of a canonical ABC transporter in a single, long polypeptide [2]. For ABCG2 (breast cancer resistance protein) the situation is more complicated – the cDNA encodes a 655 amino acid protein comprising a single N-terminal nucleotide binding domain (NBD) and a single C-terminal transmembrane domain (TMD) [3]. Dimerization of ABCG2 would be the simplest form of a functional transporter, and the protein forms a disulphide linked homodimer, but higher order aggregation states have been identified (reviewed in [4], [5].

The dimerization of ABCG2 is a critical step in its functional transport capacity [6]. Dimerization of NBDs to form a closed structure is a pre-requisite for ATP binding and hydrolysis, and pharmacological evidence has demonstrated that these steps are essential to drive affinity changes in the drug binding sites [7], [8]. Therefore, understanding the dimerization of ABCG2 is important for two reasons. Firstly, it will enable us to understand the inter-domain communication in ABCG family transporters, which are poorly understood presently [9], and which cannot be simply modelled on the better understood ABCB family transporters from mammals, and their bacterial homologues (see [1] for a discussion). Secondly, the potential of agents that prevent ABCG2 dimerization as specific inhibitors of the pump offers an avenue into drug discovery processes.

Previous studies of ABCG2 dimerization have had two principal foci. The first has been on the cysteine residues in the extracellular loop between predicted transmembrane (TM) helices 5 and 6 [10], [11], [12], [13], [14], [15]. The three cysteines in this loop have all been mutated individually, or together, and the results of these investigations demonstrated that the protein contains a single disulphide-linkage between C603 in each of two ABCG2 protomers [13], [14]. However, the disruption of this disulphide by mutation has no functional effect on the protein [10], [13], [15]. The second series of studies have aimed to identify, from bioinformatics analysis, sequence motifs in the ABCG2 TM domains that might be implicated in dimerization. For example, a GXXXG motif (where X is any amino acid, and which is a common dimerization motif in TM helices [16]) has been identified in predicted TM helix 1 but neither this, nor a more extended motif has been shown to be necessary for the formation of the ABCG2 dimer [17], [18], [19]. Additionally, a conserved residue in TM5 of ABCG2 (G553) has been found to have no role in the formation of the ABCG2 dimer [18].

**Figure 1:**
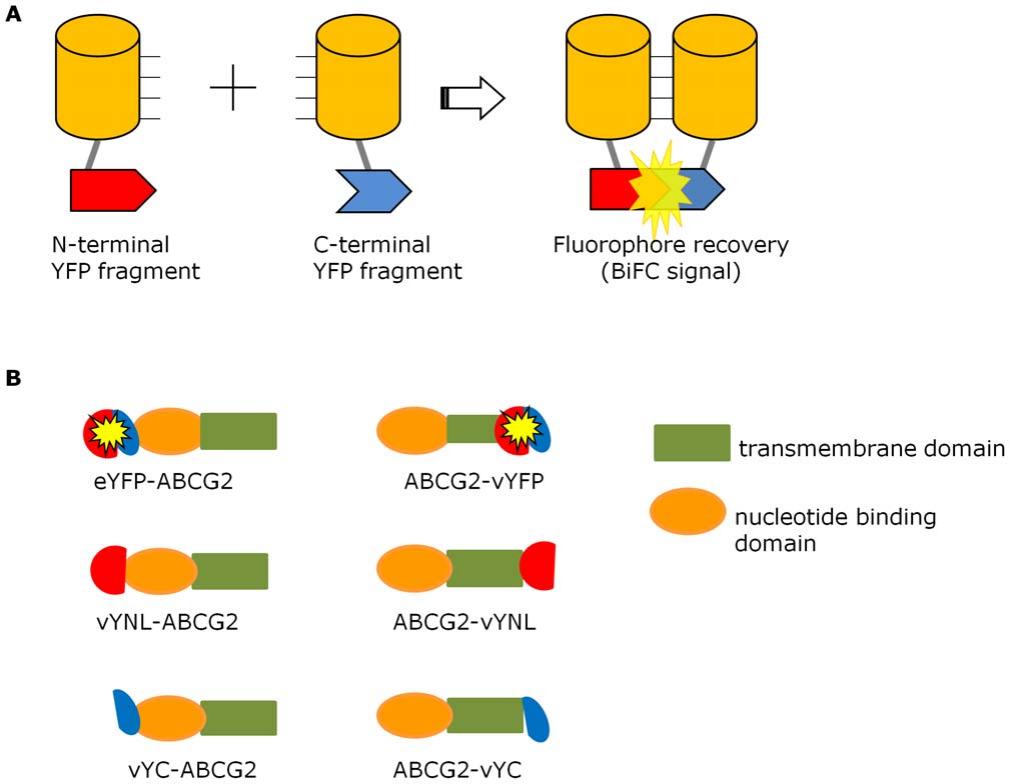
BiFC principle and ABCG2 BiFC constructs employed. **A** The underlying principle of BiFC is the refolding and maturation of a chromophore (here vYFP) that occurs upon the interaction of two proteins (here shown as cylinders) bearing complementary fragments of the YFP protein (red and blue interlocking shapes). **B** In the current manuscript, ABCG2 comprised of a N-terminal nucleotide binding domain (orange) and a C-terminal TMD (olive green) is tagged with either full length YFP variants (yellow circles), or with complementing fragments of YFP variants, shown as red and blue hemispheres. The tagging of YFP and fragments was at either the 5′ (N-terminus) or the 3′ (C-terminus) of the ABCG2 cDNA.

In the current study we investigated whether the technique of bimolecular fluorescence complementation (BiFC) can enable insights into the ABCG2 dimer. The BiFC principle (Figure 1A) is that interacting proteins tagged with molecular fragments of a β-barrel fluorescent protein (YFP etc) enable the fragments of the YFP to associate and refold, leading to the acquisition of a fluorescent entity [20]. Typically, the N-terminal fragment encodes the first 7-8 β-strands of YFP (including the tripeptide that undergoes oxidation to generate the fluorophore), whilst the C-terminal fragment encodes the latter 3-4 β-strands [21], [22]. Here, we show that ABCG2 was trafficked to the cell membrane and functional when tagged N-terminally with half-molecules of a variant form of YFP with efficient refolding kinetics (venus-YFP; vYFP [23], [24], [25], [26], and that co-expression of 2 ABCG2 constructs bearing complementing fragments of vYFP resulted in gain of vYFP fluorescence. The sensitivity of this assay was investigated by examination of ABCG2 isoforms containing point mutations that are potentially involved in dimerization. No significant differences in fluorescence complementation were observed, leading to the suggestion that the single point mutations engineered are not sufficiently altering the ABCG2 dimer to be detected, or that the BiFC technique lacks the sensitivity to detect any changes imparted by the mutations.

## Methods

### Cell culture and transfection

All tissue culture reagents were from Sigma, except for linear polyethyleneimine (PEI, from Polysciences Inc). HEK293T cells (ATCC code CRC-1573) were maintained in T75 flasks at 37°C/5% CO_2_ in Dulbecco's Modified Eagle Medium (DMEM, 4500 mg/L glucose, L-glutamine, sodium pyruvate, and sodium bicarbonate) supplemented with 10% (v/v) foetal calf serum and 100U/ml penicillin and 100 µg/ml streptomycin. Cells were passaged at 80% confluence by trypsinization. Cells were seeded 24 hours prior to transfection on 6-well (2×10^5^ cells) or 96-well (1×10^4^ cells), in reduced serum media (2-5% v/v). For immunofluorescence, cells were seeded at a slightly lower density onto poly-lysine coated glass cover slips in 6-well dishes. Cells were transfected at approximately 50% confluence using linear PEI at a PEI nitrogen : DNA phosphorus ratio of 10∶1, by addition of preformed PEI:DNA complexes to the growth media [27], [28]. Twenty-four hours after transfection media was replaced by media containing 10% v/v serum.

### Construct generation

Molecular biology reagents were from New England Biolabs unless stated. The construction of vectors encoding fragments of vYFP has been described previously [21], [23] and, in this study, ABCG2 cDNA was incorporated into these vectors by conventional restriction enzyme based cloning. ABCG2 cDNA was amplified from a pre-existing vector containing an N-terminal dodecahistidine tag, resulting in the removal of this affinity tag (primers listed in Table 1). The constructs generated are depicted schematically in Figure 1 and the following descriptors are used throughout the manuscript; vYN refers to residues 2-173 (β-strands 1–8) of venus YFP, vYC refers to residues 156–239 (β-strands 8–11)of venus YFP, vYFP refers to entire venus YFP [23], [25] and eYFP is the entirety of enhanced YFP [29].

**Table 1:** Oligonucleotide primers employed.

Primer	Sequence 5′-3′	Restriction site	Purpose
EYFPG2F	CCTGTATTTTCAGGAATTCTATGTCTTCCAG	EcoRI	Generation of ABCG2 tagged N-terminally with complete eYFP
YFPG2R	GCTTGGTACCGATCTAGAATCCAATTTAAGAATA	XbaI	Common reverse primer for tagging ABCG2 N-terminally with fragments of vYFP or with complete eYFP
VYFPG2F	CCTGTATTTTCAGGAATTCATGTCTTCCAG	EcoRI	Generation of ABCG2 tagged N-terminally with vYFP fragments
G2YFPF	CCTGTATTTTCAGGGATCCATGTCTTCCAG	BamHI	Generation of ABCG2 with C-terminal vYFP fragments
G2YFPR	GAGCTCGGATCCCTCGAGAGAATATTTTTTAAG	XhoI	Generation of ABCG2 with C-terminal vYFP fragments
E211QF	ATCTTGTTCTTGGATCAACCTACAACAGGCTTAGACTCAAG	n/a	Mutating E211Q
C603AF	ACAGGAAACAATCCGGCCAACTATGCAACATGTACT	n/a	Mutating C603A

Point mutations in vYFP-ABCG2 plasmids were introduced with pairs of oligonucleotide primers (Table 1) encoding the amino acid substitution required. Reactions conditions were as above, but contained 20 ng of template DNA, and PCR proceeded for 14–16 cycles. PCR products were treated with DpnI (10 units) by incubation for one hour at 37°C before transformation into chemically competent *E. coli* DH5α (Invitrogen). DNA sequencing validated the fidelity of mutagenesis.

### SDS-PAGE and western blotting

Cells were harvested by rinsing tissue culture plates twice with ice-cold phosphate buffered saline (PBS) supplemented with 2 mM EDTA and then collected by gentle pipetting into 1.5 ml tubes. Cells were pelleted by centrifugation at 1000 g for 5 minutes, resuspended in 250–500 µl of ice-cold PBS/2 mM EDTA containing EDTA-free protease inhibitor cocktail set III (Calbiochem) and then lysed by sonication (2×10 second bursts, 40% output Microsonics Instruments). Protein concentration was determined by a modified Lowry assay (Bio-Rad) and equal quantities of protein were loaded onto 10% w/v polyacrylamide gels as previously described. Protein gels were stained with Coomassie stain (0.01% (w/v) Coomassie blue R250, 10% (v/v) acetic acid, 40% (v/v) methanol) and de-stained in acetic acid/methanol (both 10% v/v) overnight. For western blotting, proteins were transferred onto nitrocellulose membrane (Amersham) by electrophoresis in 25 mM Tris, 192 mM glycine. Membranes were then incubated in a blocking solution, (5% skimmed milk powder (w/v) in TBST (25 mM Tris, 0.15 M NaCl, pH 7.6, 0.1% (v/v) Tween 20)) for one hour at room temperature. Membranes were then incubated with primary antibody (BXP-21 (Calbiochem) at 1∶2000 or anti-GFP (Roche) at 1∶1000 dilutions) prepared in the same blocking solution overnight at 4°C. After washing, membranes were incubated in secondary antibody, (rabbit anti-mouse horseradish peroxidase (DAKO) diluted at 1∶2000 in blocking solution) for 1 hour at room temperature. The membranes were then washed and developed using Enhanced Chemiluminescence, ECL (Supersignal West Pico; Thermo Scientific).

### Flow cytometry

Transiently transfected HEK293T cells were analysed by flow cytometry for both protein expression and protein function in accordance with previous protocols [30]. Cells were washed twice then harvested in sterile, ice-cold PBS and collected into 15 ml Falcon tubes. Cells were pelleted by centrifugation at 150 g for 5 min at 4°C then washed with ice-cold PBS and re-centrifuged. Pellets were resuspended in flow cytometry buffer (1% (v/v) foetal calf serum in phenol red-free DMEM) and aliquotted into facs tubes as 100 µl aliquots at a cell density of 1–2×10^7^ cells per ml. For detection of cell surface ABCG2 expression, transfected HEK293T cells were incubated with 1∶100 dilution of a phycoerythrin (PE; λ_EX_ 546 nm; λ_EM_ 578 nm) conjugated monoclonal antibody (5D3; R&D systems). An isotype control antibody IgG-PE (Abcam) was employed in parallel experiments to validate the specificity of 5D3 binding. For analysis of function, cells were incubated with mitoxantrone (λ_EX_ 635 nm; λ_EM_ 670 nm; 5 mM; Sigma) in the presence or absence of fumitremorgin C (FTC, a gift of the NIH, Maryland; 1–5 mM). Substrates and inhibitors were added from DMSO stocks so that the final solvent concentration was below 0.5% v/v. After 30 minutes incubation at 37°C with occasional inversion cells were pelleted by centrifugation, washed twice with ice-cold flow cytometry buffer and finally resuspended in 300–400 µl of flow cytometry buffer. The tubes were then analysed by Beckman-Coulter XL-MCL Flow cytometer. Flow cytometry data were analysed using WEASEL v.2 (The Walter and Eliza Hall Institute of Medical Research). Cells were gated using forward scatter (FS) and side scatter (SS) to exclude dead cells, cellular debris etc. Gated cells were then quantified as histograms representing the number of cell events with a particular fluorescence.

### Immunofluorescence

HEK293T cells transfected with required constructs were fixed on cover slips with 4% (w/v) paraformaldehyde (PFA) in PBS for a maximum of 5 minutes at room temperature before being washed twice with PBS and incubated in 3% (w/v) BSA in PBS (blocking solution) for one hour at room temperature. For cells that need permeabilization (to allow entry of an antibody that recognizes an intracellular epitope, e.g. BXP-21) a 5 minute incubation in 0.05% (v/v) TritonX-100 in PBS was included before the blocking step. Following blocking, cells were incubated for 1 hour with primary antibody (BXP-21; Calbiochem, or 5D3; Millipore) prepared at 1∶2000 dilution in incubation buffer (0.3% w/v BSA in PBS). The primary antibody solution was removed and the cells were washed thrice with incubation buffer. Cells were then incubated in secondary antibody (goat anti-mouse monoclonal antibody conjugated to AlexaFluor 488 green fluorescent dye; Invitrogen), at a 1∶1000 dilution in incubation buffer. Cells were then washed thrice with incubation buffer and then mounted onto microscopic slides with 20–40 µl of Vectashield Mounting Medium with DAPI (Vector labs).

### Confocal microscopy

Greiner Bio-one ‘μclear’ black-sided 96-well plates (655090) were coated with poly-L-lysine for an hour at room temperature, before seeding HEK293T cells at a density of 1.0–1.5×10^4^ cells per 100 µl of medium per well. Cells were transiently transfected, as described earlier, and 48 hours subsequently, cells were washed twice with sterile HEPES buffered saline (HBS) and then maintained in HBS with membrane permeant Hoechst 33342 dye (0.4 µM) added to stain nuclei before being analysed on an ImageXpress Micro imaging system, using a Nikon 40x NA 0.6 Extra Long Working Distance objective (Molecular Devices, Inc. USA, [23]). Cells were imaged (9 sites/well) using the following excitation (λex) and emission (λem) filter sets to detect (i) Hoechst 33342 λex 377 nm (bandpass; BP 50 nm) and λem 447 nm (BP 60 nm) and (ii) YFP BiFC, λex 482 nm (BP 35 nm) and λem 536 nm (BP 40 nm). An automated cell scoring algorithm applied to each image (MetaXpress 2.0, Molecular Devices) identified the total number of cells by nuclear count (Hoescht staining), and the percentage of BiFC “positive” cells, based on a manual minimum threshold for average cytoplasmic intensity of YFP BiFC fluorescence (set with reference to positive/negative plate controls). The individual cell data was then filtered by AcuityXpress 1.0 (Molecular Devices) to obtain the average cytoplasmic BiFC intensity for the “positive” transfected population. Typically, several thousand cells were identified and quantified for each condition in each independent experiment.

### Statistical analysis

Data were analyzed with Prism5 (Graph Pad) and/or Microsoft Excel, using ANOVA and Student's t-tests, with P-values of less than 0.05 being considered significant for any set of data.

## Results and Discussion

### N-terminal but not C-terminal tagging of ABCG2 with YFP fragments supports protein localization and function

The dimerization of ABCG2 was investigated using bimolecular fluorescence complementation, a protein:protein interaction technique that relies on the ability of molecular fragments of YFP to re-associate and refold into a fluorescent structure (Figure 1A [31]). Here we employed the vYFP isoform as its BiFC fragments can refold at 37°C with fast folding kinetics [22], [23], [24], [26]. ABCG2 has both its N-terminus and its C-terminus exposed to the intracellular surface [32] and thus 6 constructs were required for use in this study (Figure 1B). Two of these, YFP-ABCG2 and ABCG2-YFP, are controls in which the full length YFP is tagged to the N- or C-terminus respectively of ABCG2. For convenience in these control constructs, the N-terminal full length construct employed was eYFP, rather than vYFP. The other 4 constructs represent proteins in which residues 2–173 (vYN) and residues 156–239 (vYC) are tagged to the N-terminus of ABCG2, and the same two fragments fused to the C-terminus of ABCG2. All constructs are shown diagrammatically in Figure 1B, and the associated nomenclature is also given in this figure.

**Figure 2:**
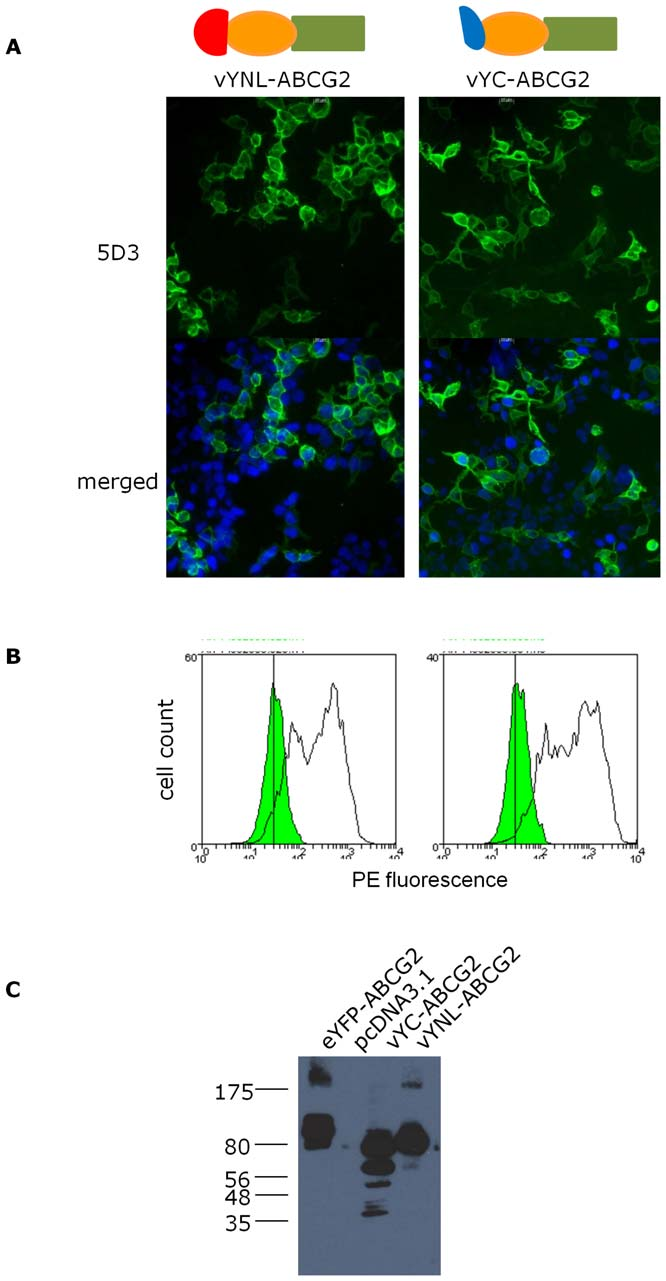
N-terminal tagging with ½ YFP molecules does not affect expression and targeting of the ABCG2 protein to the plasma membrane. **A** HEK293T cells were transfected as described and 48 hours later were fixed and incubated with monoclonal 5D3 antibody directed towards an extracellular epitope in ABCG2, and a secondary antibody coupled to AlexaFluor 488, before being counter-stained with DAPI and mounted for fluorescence microscopy. Top panels show the presence of ABCG2 at the plasma membrane, and the lower panel is a merged image of the 5D3 and DAPI. **B** Cells were transfected and analysed by flow cytometry as described following incubation with phycoerythrin (PE) conjugated control antibody (IgG-PE; green filled distribution) or PE-conjugated 5D3 (unfilled distribution) which recognizes an extracellular epitope of ABCG2 In **A** and **B** the left hand column represents ABCG2 tagged N-terminally with vYN (residues 2–173 of vYFP), the right hand panel represents ABCG2 tagged N-terminally with vYC (residues 156–239 of vYFP). Scale bars are 10 µm, and the data are representative of at least 4 independent transfections. C Cells were transfected and 20 µg of whole cell lysate was analysed by SDS-PAGE and western blotting, using BXP-21 to identify ABCG2 fusion proteins. The expression level of the three constructs is comparable. A number of lower molecular weight breakdown products were observed for the fusion with the C-terminus of vYFP, although the extent of this was variable. A parallel Coomassie stained gel was employed to verify equal protein loading (data not shown).

All constructs were examined for expression following transient transfection of single constructs into HEK293T cells and, using polyethyleneimine (PEI) as the transfection reagent [27], the percentage transfection efficiency was routinely greater than 50%, in accordance with previous results on other ABC proteins expressed in HE293T cells [33]. To validate the cell surface expression of ABCG2 isoforms carrying vYFP fragments at the N-terminus we performed immunofluorescence on fixed, but unpermeabilized cells with the monoclonal antibody 5D3, which recognizes an extracellular epitope ([34], Figure 2A). Parallel flow cytometry with the same antibody again revealed the surface expression of ABCG2 and confirmed the high percentage transfection efficiency. Western blotting under denaturing conditions using the BXP-21 antibody [35] revealed the presence of proteins with the expected molecular weights (vYN-ABCG2 at approximately 90 kDa; and vYC-ABCG2 at approximately 80 kDa), with evidence of lower molecular weight breakdown products, presumably reflecting the increased protease sensitivity of ABCG2 when tagged with partially unfolded protein fragments of vYFP (as discussed at length in [26]).

**Figure 3:**
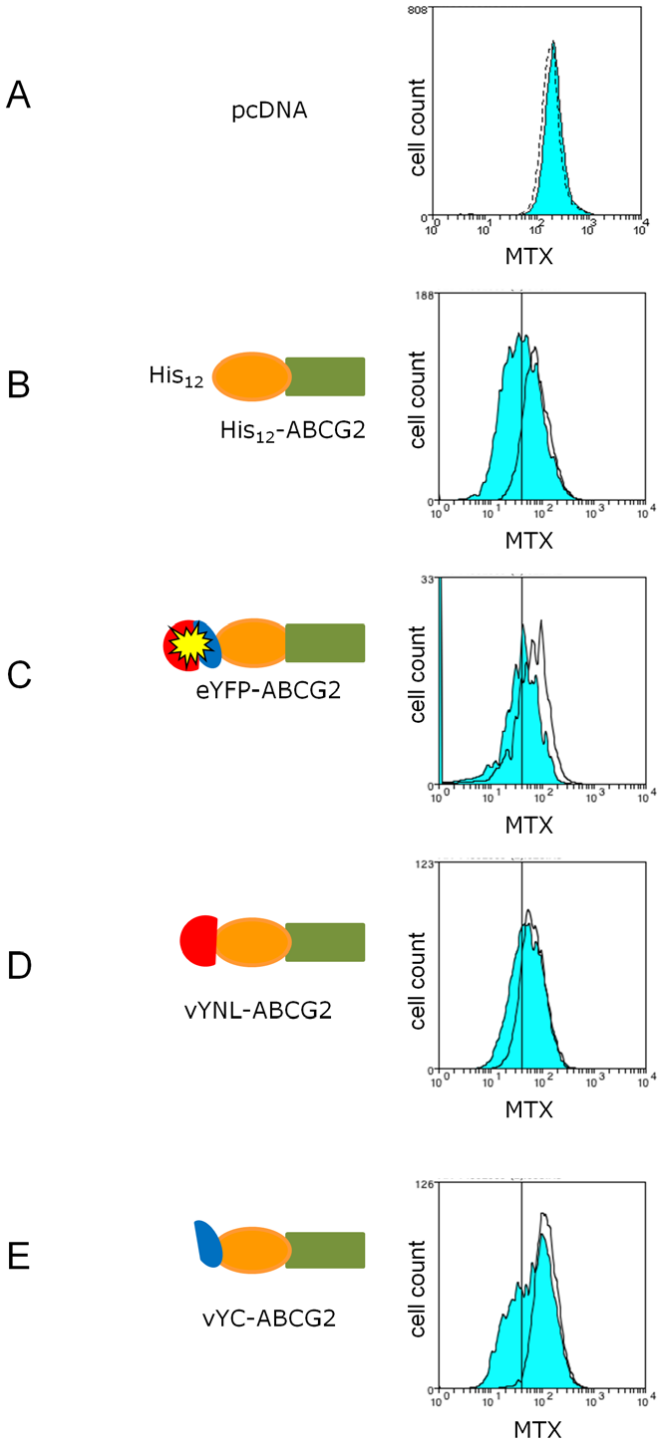
N-terminal tagging with ½ YFP molecules does not affect function of the ABCG2 protein. HEK293T cells were transiently transfected with YFP_ABCG2 constructs (C-E) or negative control (empty pcDNA vector; A) and positive control (His_12_-ABCG2) as described in Methods and Materials. Following transfection aliquots of cells were incubated with the ABCG2 substrate mitoxantrone in the presence or absence of the inhibitor fumitremorgin C (FTC) and cellular fluorescence determined by flow cytometry. Blue filled histograms represent the cellular fluorescence in the absence of FTC, and the rightward shift in the presence of the inhibitor (black lines) demonstrates functional competence at a level similar to those of the characterized control His_12_-ABCG2 (B). The apparent large number of cells with zero fluorescence in the eYFP-ABCG2 panel (C) is a fluorescence artefact due to interference from the YFP fluorescence of this construct. The graphs are representative of >5 independent experiments.

The effect of tagging ABCG2 at the N-terminus has been previously investigated with poly-histidine [8], [36] and full length green fluorescent protein [6] and in both cases the tagged protein retained function. However, the effect of tagging with fragments of YFP has not previously been described. We examined the ability of vYN-ABCG2 and vYC-ABCG2 to export mitoxantrone in transiently transfected cells using flow cytometry (Figure 3), using the other tagged proteins mentioned previously as internal controls. Both constructs displayed an export of mitoxantrone from cells that was inhibited by fumitremorgin C (FTC), a specific inhibitor of ABCG2 [37]. Although in these experiments the inhibitory effect of FTC is modest in comparison to other studies of ABCG2 isoforms [38], we have shown here that data obtained with our YFP and half-YFP tagged constructs (Figure 3 C-E) is similar to that of the wild type ABCG2 construct (Figure 3B), supporting the conclusion that that the N-terminal tagging of ABCG2 with half molecules of YFP does not negatively impact on the function of the protein.

In contrast to the data shown in Figure 2 and 3, the tagging of ABCG2 at the C-terminus with half molecules of YFP (or with full length YFP) resulted in poor trafficking, increased intracellular retention, and no convincing functional mitoxantrone export (data not shown). Previously, the final few C-terminal residues of ABCG2 have been shown to be sensitive to mutation (in terms of protein trafficking [39]) and this further indicates that the C-terminus contains the sequence motifs that are recognized by cellular processing machinery.

### Co-expression of ABCG2 bearing complementing halves of venus YFP results in BiFC at the plasma membrane

**Figure 4:**
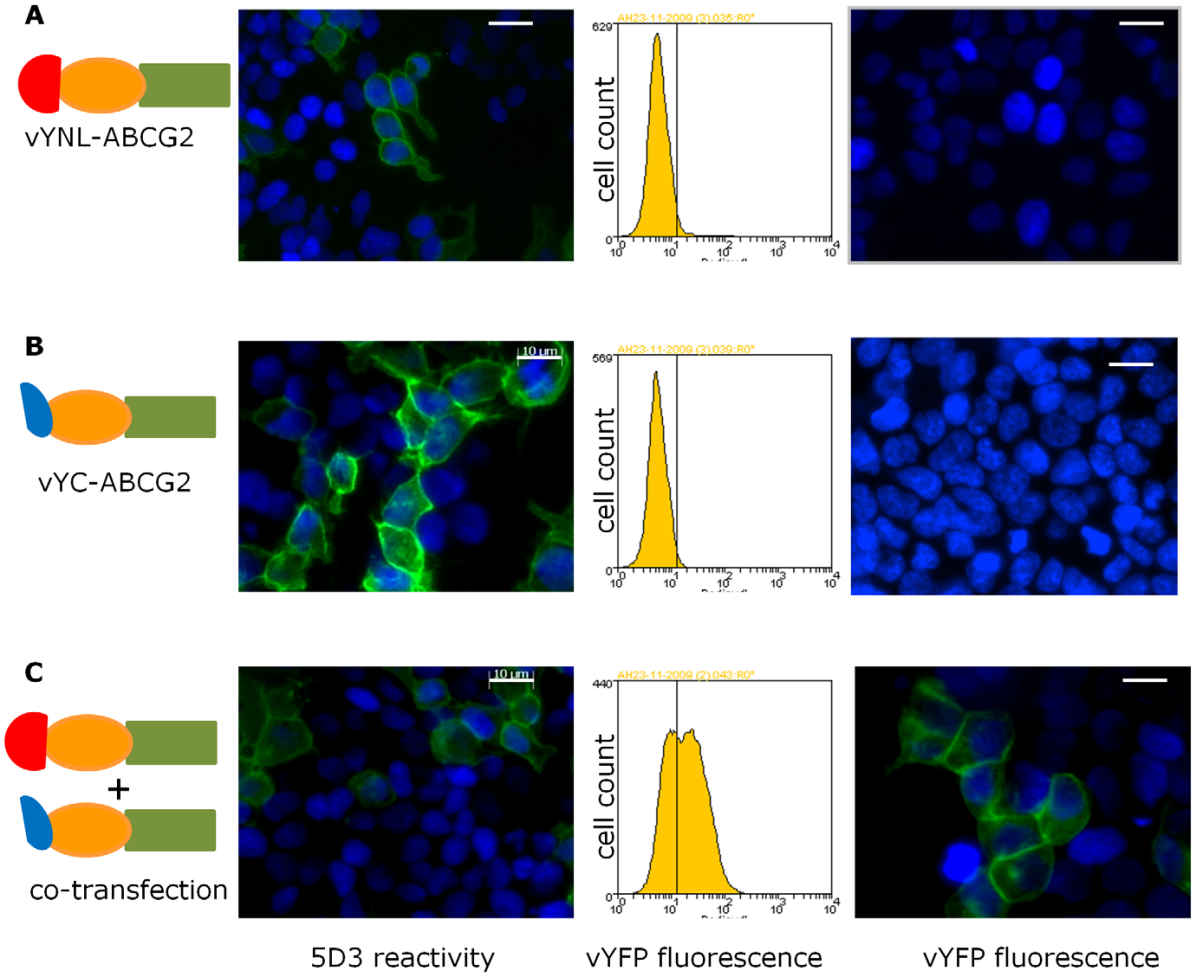
Complementation of YFP-ABCG2 constructs occurs at the plasma membrane. Constructs expressing half-tagged vYFP-ABCG2 individually (**A, B**) or together (**C**) were transfected into HEK293T cells as indicated schematically to the left. Constructs reached the plasma membrane as confirmed by immuno-staining with 5D3 antibodies (left hand panel). No fluorescence could be detected at 515 nm by flow cytometry of cells transfected with the vYN-ABCG2 or vYC-ABCG2 construct, but in co-transfected cells a YFP fluorescence was detected (middle panel). Confirmation of the BiFC interaction was obtained from imaging fixed cells by fluorescence microscopy (right hand panel). DAPI was employed as a nuclear counter-stain. Individual transfections resulted in only DAPI labelled nuclei in such experiments. Data are representative of at least 4 independent experiments.

Having compared the effects of N-terminal and C-terminal tagging of ABCG2, only N-terminal tagged isoforms of ABCG2 were investigated for their ability to promote vYFP fragment association and refolding. To validate BiFC we performed both single transfections and co-transfections with vYN-ABCG2 and vYC-ABCG2 (Figure 4). As expected, single transfections into HEK293T cells did not result in the detection of a YFP fluorescent signal (Figure 4A,B, middle and right hand panels), although protein expression was confirmed in both cases (Figure 4A, left panel). The co-transfection of both constructs resulted in protein expression at the membrane as detected by 5D3 antibody (Figure 4C, left panel), and in the formation of a fluorescence complementation signal detectable by both flow cytometry and by fluorescence microscopy (Figure 4C, middle and right panels). Time course experiments revealed that this complementation could be observed as early as 16 hours post-transfection at 37°C, but that optimal signals were obtained 36–48 hours post-transfection.

### ABCG2 BiFC with other non-specific partner proteins results in aggregations of BiFC complexes

**Figure 5:**
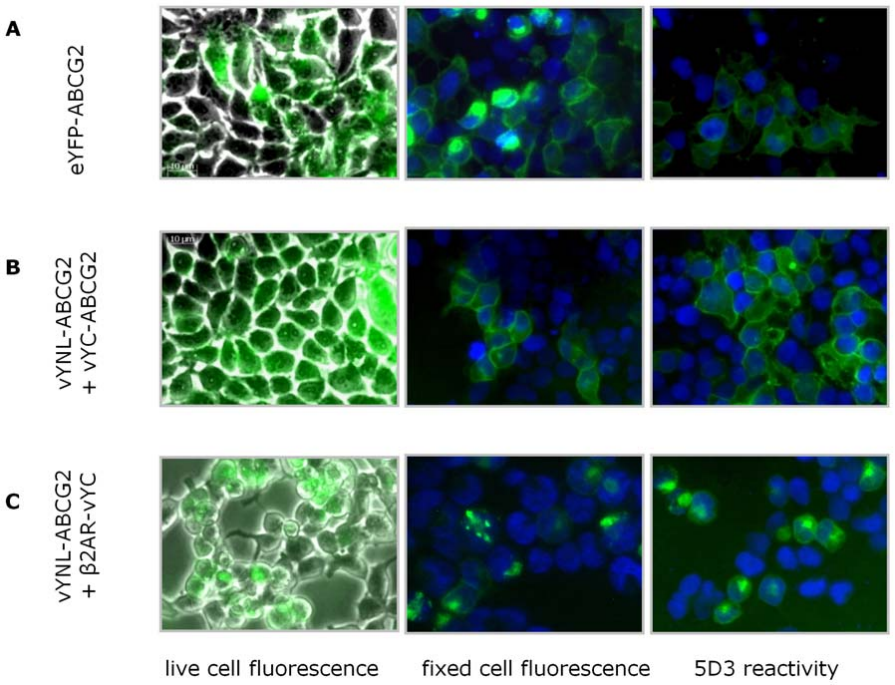
ABCG2 bimolecular fluorescence complementation is specific. HEK293T cells were transfected with constructs expressing either eYFP-ABCG2 (as a positive fluorescence control, **A**), complementing halves of vYFP tagged to ABCG2 (**B**) or with vYN-ABCG2 and a non-specific interaction partner β2 adrenergic receptor (β2AR) with a C-terminal vYC fragment (**C**). Live cell imaging (left hand panel) shows that all three transfections result in cellular fluorescence indicating the complementation of both the cognate (ABCG2:ABCG2) and non cognate (ABCG2: β2AR) protein pairs. However, cells fixed and counter stained with DAPI (middle panel) show that the BiFC signal arising from ABCG2:ABCG2 interaction is membrane localized, in contrast to the BiFC signal from the ABCG2: β2AR interaction which shows cytoplasmic retention. Immunofluorescence of cells with anti ABCG2 antibodies (right panel) further demonstrates that the ABCG2: β2AR interaction results in intracellular retention of ABCG2. Data are representative of at least 4 independent experiments.

The interaction of vYN and vYC to form a fluorescent complex is essentially irreversible, as numerous hydrogen bonds are formed during the association, and the strand arrangement of the YFP β-barrel precludes a simple dissociation of the two fragments [21], [22], [23]. Consequently, it is important in BiFC studies to determine whether the driving force for complementation is the interaction of the protein partners, or whether the vYN:vYC interaction is the driving force [22], [23], [24], [26]. To address this issue we performed co-expression studies in which the cognate interaction of two ABCG2 proteins (i.e. vYN-ABCG2 and vYC-ABCG2) was examined in parallel with co-transfections in which vYN-ABCG2 was expressed together with a non-specific partner protein, namely the β2 adrenergic receptor (β2-AR, [21]). Co-expression of vYN-ABCG2 and vYC-ABCG2 resulted in a fluorescence complementation signal that was at the cellular surface (Figure 5B, middle and right hand panel), indicating that the BiFC interaction did not detract from the effective trafficking of ABCG2. Conversely, the interaction of vYN-ABCG2 and β2-AR-vYC resulted in the observation of dense cytoplasmic fluorescence foci, and the retention of ABCG2 within the cell (Figure 5C, middle and right hand panel; this non-specific BiFC was also associated with increased cell death). Thus, although there is evidence of a BiFC interaction observed in these latter transfectants, the interaction of ABCG2: β2AR is not conducive to efficient protein trafficking. This type of observation has been made previously in studies of B cell receptor oligomers [40], where specific protein:protein interactions between partner proteins resulted in membrane localized BiFC, whereas non-specific interactions resulted in cellular accumulation of BiFC fluorescence.

### Analysis of predicted ABCG2 dimer interface mutations by BiFC

Understanding the dimer interface of ABCG2 is central to understanding its mechanism. We investigated whether residues in ABCG2, which are likely to participate in interactions between two monomers in a dimer, could result in a change in the dimerization reported by BiFC. Mutation of the conserved Walker-B glutamate residues (in ABCG2 this corresponds to the mutation E211Q) has been shown in numerous *in vitro* and structural investigations of other ABC proteins to result in the tighter apposition of the 2 NBDs with concomitant, irreversible trapping of the nucleotide substrate [41], [42], [43], [44], [45]. A second mutation, C603A, was introduced to prevent the inter-dimer disulphide bond that has been shown to be necessary for homodimer formation on non-denaturing SDS-PAGE, but which is not required for function [13], [15].

**Figure 6:**
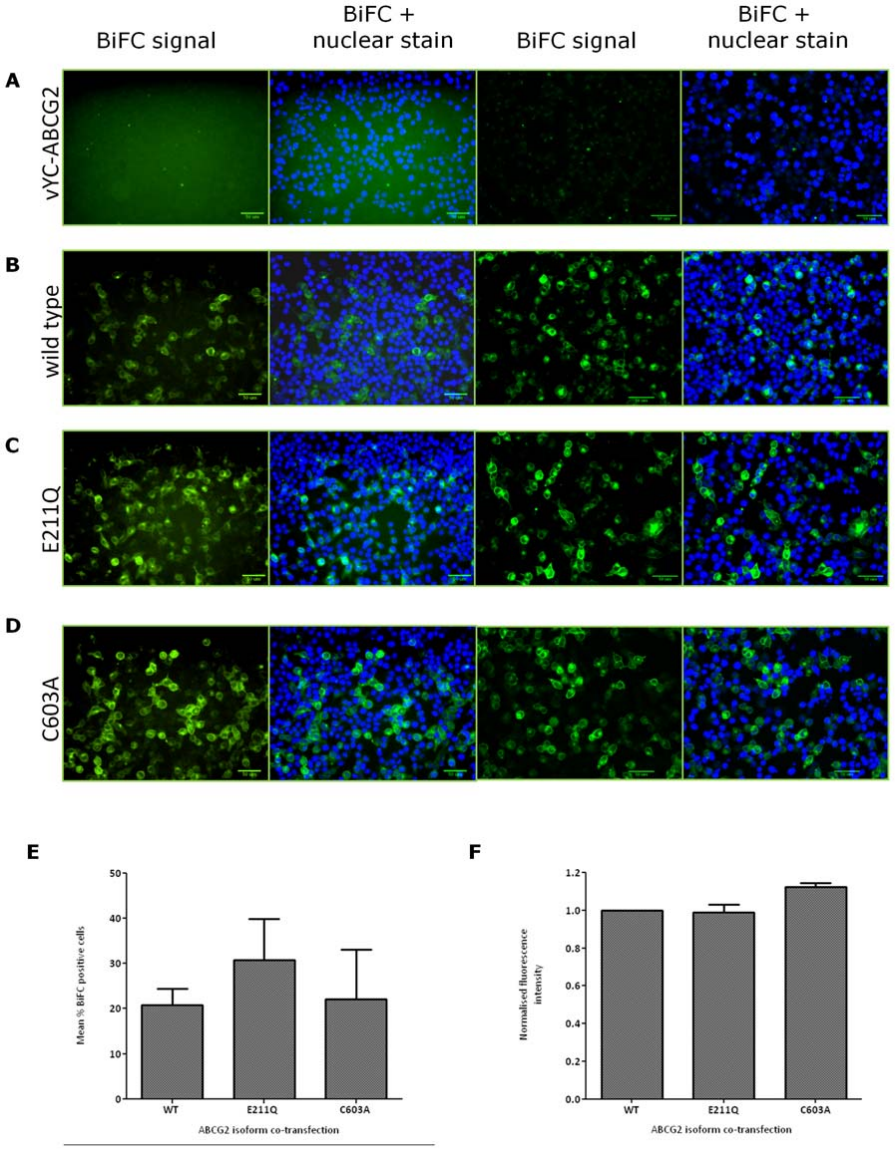
ABCG2 BiFC quantification by high content screening. HEK293T cells were transfected in 96-well plates as described in the methods, with pairs of constructs expressing vYN-ABCG2 and vYC-ABCG2 (panels **B, C, D**) or with a single construct expressing only vYC-ABCG2 (**A**). ABCG2 isoforms expressed were wild type (**A, B**), E211Q (**C**) and C603A (**D**). For each transfection two representative images from multi-well confocal acquisition are shown, with and without the Hoechst 33342 stained nuclei for clarity. Images were taken with a 40X objective on a Molecular Devices IX Micro plate reader. **E** The percentage BiFC positive cells was determined as described in the Methods, employing negative (single transfections) to set thresholds for fluorescence determination above background. **F** Mean intensity fluorescence levels were detected for YFP complementation for each ABCG2 isoform, and the data was normalised within each experiment to the mean fluorescence intensity observed for wild type ABCG2 BiFC. Data in **E** and **F** are the mean (± s.e.m.) of 4-7 independent experiments.

We co-transfected ABCG2 wild type and mutant isoforms tagged with N- and C-terminal fragments of vYFP (Figure 6A-D). All three constructs (WT, E211Q and C603A) showed BiFC (Figure 6B-D respectively) producing a fluorescence signal far in excess of the background observed with a single transfection (Figure 6A). The use of 96-well plate transfections and quantitative image analysis software (see Methods) enabled us to determine two parameters that could reflect altered dimerization between wild type ABCG2 and mutant isoforms. Firstly, we determined the percentage of cells with fluorescence above a background threshold (Figure 6E). Although the percentage of cells showing BiFC was greater for the E211Q mutant – which might be expected to form a stronger dimer – this was not statistically significant by ANOVA. Secondly, we quantified the mean fluorescence intensity signal as a measure of the strength of the BiFC interaction (Figure 6F). Again, analysis of several thousand cells in several independent transfections showed that there was no difference in the fluorescence intensity for either mutant ABCG2 isoform, compared to the wild type.

This data indicates that the initial association of the interacting monomers – which is the event that BiFC “captures” - is not sufficiently altered by either of the mutations we introduced to be detected. For the Walker-B mutation, structural data on related ABC proteins places the Walker-B glutamate residues within 8 Å of the opposite NBD [46], and it has been shown on several occasions that the mutation of the conserved glutamate to glutamine produces an NBD protein that *in vitro* is able to associate into a stable dimeric state in the presence of ATP, which occludes ATP (see references in [46]) It may be that in the current experiments in intact cells this tight NBD dimerization is not recapitulated. For cysteine-603, which is located in the extracellular loop between TM5 and TM6, it is arguable that our data is consistent with the hypothesis that the residue is not essential for dimerization [13], [15] [38].

It seems probable that our inability to detect changes in BiFC signal with these two mutations reflects both a combination of the irreversibility of BiFC following initial association, which has been discussed previously [21], [26], and the potentially minor alterations to ABC2 dimer status that a single amino acid change on a large protein:protein interaction surface would engender. Despite recent improvements to the specificity of the BiFC interaction which may reduce false positive interactions (e.g. [24], [26]), the irreversibility is still unaddressed, precluding us from being able to use BiFC to further investigate the structural basis of ABCG2 homodimerization, i.e. identifying individual residues at the dimer interface. However, it remains possible that the effects of multiple mutations to a predicted interfacial site could be detected by BiFC, e.g. the surface of helix TM1 which contains a T402L/G406L/G410L dimerization motif [17].

Bimolecular fluorescence complementation is a powerful technique for the detection of protein:protein interactions which may be homo- and/or hetero-dimeric in nature, including previous studies of cuticular lipid ABCG-type transporters in plants [47]. We have demonstrated here that a BiFC signal, specific to homodimeric ABCG2 interaction at the plasma membrane, was observed and, importantly, that tagging ABCG2 with molecular fragments of vYFP did not perturb function or localization. Our work, and the recent demonstration that ABCG2 can also be studied by Förster resonance energy transfer (FRET; [38]), should promote further studies to examine heteromeric interactions of ABCG2, such as those with the regulatory PIM1 kinase [48], using these fluorescence technologies.
